# New York Ophthalmic School

**Published:** 1863-04

**Authors:** 


### NEW YORK OPHTHALMIC SCHOOL.

The New York Ophthalmic School and Hospital held its Eleventh Anniversary on the 24th ult., in the Medical College in Fourteenth St., before a large and highly intelligent audience. The exercises of the evening were introduced with prayer by the Rev. Edward Thomson, M. D., Editor of the Christian Advocate and Journal. The names of the graduating class were read by Dr. Mark Stephenson, lecturer on the anatomy, pathology and treatment of diseases of the Eye, who remarked to the President that these young gentlemen were students from the different medical colleges in this city; that they have attended his lectures on ophthalmic surgery; been examined every Saturday by Dr. M. P. Stephenson; attended the cliniques three days in the week; learned to diagnose diseases of the eye; watched the effect of remedies from day to day, and witnessed the various operations at the hospital, where over one thousand patients are prescribed for every year. And he further remarked he thought they were better qualified to practice this department of their profession than many physicians who have been twenty or thirty years in practice.

The diplomas were presented by Solomon Jenner, A. M., President of the Institution, as follows:

A. E. Jenner, M. D., Ohio; J. M. Waddle, Yates County, N. Y.; J. P. Schenck, jr., Duchess County, N. Y.; De Witt Webb, Duchess County, N. Y.; J. H. McCann, M. D., Louisville, Ky.; Robert King, Geneva, N. Y.; H. G. Olmsted, M. D., N. Y. City; James Hutchinson, St. John, N. B.; Charles P. Sanderson, M. D., Ohio; R. J. Mordon, M, D., Canada West; J. H. Chittenden, Binghamton, N. Y.; Thomas Thompson, Delaware Co., N. Y.; G. A. Hayunga, M. D., Canada West; J. H. Hunter, M. D., Concord, N. H. ; M. C. Rowland, M. D., Washington County, N. Y.; W. J. Orton, Broome Oounty, N. Y.

The graduates were then addressed in a very forcible and appropriate manner by Dr. Marcus P. Stephenson, one of the attending surgeons, under whose immediate instruction and examination they had been during the past winter. The Valedictory Address was delivered in a very able and pleasing manner by Alexander E. Jenner, M. D., one of the graduating class, and the exercises of the meeting closed with an eloquent address by J. P. Garrish, M. D.

